# European HPC cloud infrastructure for managing SARS-CoV-2 data in compliance with GDPR

**DOI:** 10.1093/eurpub/ckac129.427

**Published:** 2022-10-25

**Authors:** C Dellacasa, M Ortali, E Rossi, M D'Antonio, T Osmo, F Prasser, M Puskaric, E Rinaldi, Orchestra Working Group, G Scipione

**Affiliations:** 1 HPC Department, CINECA Consorzio Interuniversitario, Casalecchio di Reno, Italy; 2 CINES Centre Informatique National de l'Enseigneme, Montpellier, France; 3 High Performance Computing Center Stuttgart (HLRS), Stuttgart, Germany; 4 Berlin Institute of Health (BIH), Charité, Berlin, Germany; 5 ORCHESTRA Consortium, Verona, Italy

## Abstract

The Connecting European SARS-CoV-2 Cohorts to Increase Common and Effective Response to SARS-CoV-2 Pandemic (ORCHESTRA) consortium, led by University of Verona (Italy), brings together key European academic experts and research institutions in infectious diseases, data management and High Performance Computing (HPC) from 26 organizations (extending to 37 partners) from 15 countries. The project aims to create a new pan-European cohort built on existing and new large-scale population cohorts in European and non-European countries to significantly impact on the responsiveness to SARS-CoV-2. The integration and analysis of the very heterogeneous characteristics of SARS-CoV-2 data coming from many different sources such as EHR, retrospective and prospective patient registries, and related ‘omics’ data (incl. genomics, proteomics and transcriptomics) can benefit of data analytics enabled by HPC, where both high compute performance and fast storage capabilities are immensely important. During the first year of the project, a dedicated HPC cloud infrastructure have been designed and partially deployed to fulfill the functional requirements for data management ensuring healthcare data confidentiality/privacy, integrity and security in compliance with the European GDPR regulations. The result is an infrastructure for Data Management composed by three main layers: National Data Providers; National Hubs (one for each HPC center involved: CINECA - Italy, CINES - France and HLRS - Germany), to centralize data at national level and to support data storage, sharing and analysis on data ingested from the National Data Providers; ORCHESTRA Data Portal: the pan-European portal for sharing aggregated data and results. Currently data collection is on going; at the end of the project, clinical centers are expected to have enrolled more than 10.000 patients with about 50.000 samples for the prospective studies.

**Key messages:**

• The SARS-CoV-2 crisis made evident the need to manage and analyse very heterogeneous health data coming from many different resources across different countries.

• The HPC cloud infrastructure released for the Orchestra project can act as a model to manage future public health threats.

